# Eicosapentaenoic Acid Protects against Palmitic Acid-Induced Endothelial Dysfunction via Activation of the AMPK/eNOS Pathway 

**DOI:** 10.3390/ijms150610334

**Published:** 2014-06-10

**Authors:** Che-Hsin Lee, Shin-Da Lee, Hsiu-Chung Ou, Su-Chuan Lai, Yu-Jung Cheng

**Affiliations:** 1 Department of Microbiology, School of Medicine, China Medical University, 40402 Taichung, Taiwan; E-Mail: chlee@mail.cmu.edu.tw; 2 Department of Physical Therapy and Graduate Institute of Rehabilitation Science, China Medical University, 40402 Taichung, Taiwan; E-Mails: shinda@mail.cmu.edu.tw (S.-D.L.); ouhc@mail.cmu.edu.tw (H.-C.O.); miracle0820@gmail.com (S.-C.L.)

**Keywords:** eicosapentaenoic acid, palmitic acid, endothelial dysfunction, AMP-activated protein kinase

## Abstract

Recent studies have shown that free fatty acids are associated with chronic inflammation, which may be involved in vascular injury. The intake of eicosapentaenoic acid (EPA) can decrease cardiovascular disease risks, but the protective mechanisms of EPA on endothelial cells remain unclear. In this study, primary human umbilical vein endothelial cells (HUVECs) treated with palmitic acid (PA) were used to explore the protective effects of EPA. The results revealed that EPA attenuated PA-induced cell death and activation of apoptosis-related proteins, such as caspase-3, p53 and Bax. Additionally, EPA reduced the PA-induced increase in the generation of reactive oxygen species, the activation of NADPH oxidase, and the upregulation of inducible nitric oxide synthase (iNOS). EPA also restored the PA-mediated reduction of endothelial nitric oxide synthase (eNOS) and AMP-activated protein kinase (AMPK) phosphorylation. Using AMPK siRNA and the specific inhibitor compound C, we found that EPA restored the PA-mediated inhibitions of eNOS and AKT activities via activation of AMPK. Furthermore, the NF-κB signals that are mediated by p38 mitogen-activated protein kinase (MAPK) were involved in protective effects of EPA. In summary, these results provide new insight into the possible molecular mechanisms by which EPA protects against atherogenesis via the AMPK/eNOS-related pathway.

## 1. Introduction

Accumulated saturated free fatty acids are known to induce endothelial dysfunction that leads to atherosclerosis. Free fatty acids trigger endothelial apoptosis and inhibit cell cycle progression [1]. The atherosclerotic process is associated with a decrease in the bioavailability of nitric oxide (NO) from endothelial cells, which constitutively express enzyme endothelial nitric oxide synthase (eNOS). Free fatty acids impair insulin-mediated vasodilation and eNOS activation [2,3] and reduce NO production [4]. Additionally, excessive free fatty acids stimulate the generation of free radicals, which attack important macromolecules and lead to cell damage and homeostatic disruption [5]. Therefore, therapeutic interventions that reduce free fatty acid-induced endothelial apoptosis and reductions in eNOS may prevent the development of atherosclerosis. Palmitic acid (PA) is the main saturated free fatty acid in the bloodstream. The exposure of endothelial cells to PA leads to cell necrosis [6] and the release of inflammatory cytokines IL-6 [7]. Moreover, palmitate can induce apoptosis in cultured bovine retinal pericytes via increases in oxidative stress [8]. Thus, endothelial cell exposure to PA is a suitable model for the exploration of the mechanisms by which free fatty acids induce endothelial dysfunction.

Eicosapentaenoic acid (EPA) is abundant in fish oil and is a polyunsaturated fatty acid of the ω-3 series. Studies have shown that ω-3 polyunsaturated fatty acid has beneficial effects in atherosclerosis [9]. The intake of EPA can reduce the risks of cardiovascular diseases, hypertriglyceridemia, hypertension and inflammation [10,11,12]. The incubation of endothelial progenitor cells with EPA significantly increases cell numbers, colony forming units and EPC migratory capacities [13]. In human endothelial cells, EPA can enhance the production of the endothelium-derived relaxing factor, nitric oxide (NO), via the upregulation and activation of endothelial nitric oxide synthase [14], which might improve the function of the vascular endothelium [15]. Although numerous studies have used EPA to prevent or treat atherosclerosis [16,17], it remains unclear whether EPA can protect against endothelial damage from free fatty acids. In this study, we used PA to examine whether EPA could protect the endothelium from free fatty acid-induced damage. Changes in oxidative stress, cell apoptosis, eNOS and AMPK were explored. 

## 2. Results

### 2.1. EPA Blocked Cell Death Induced by PA in HUVECs

**Figure 1:**
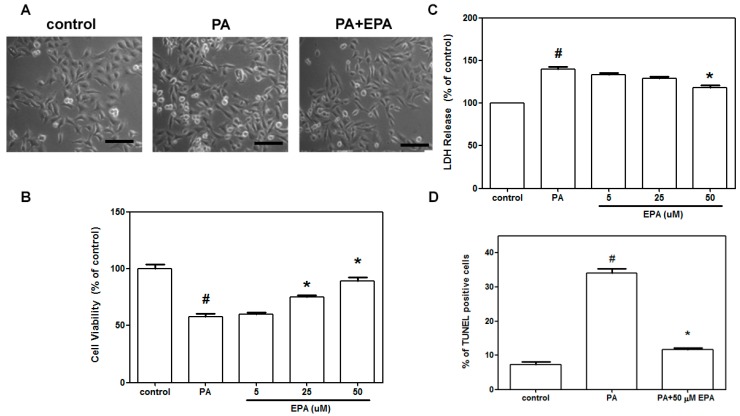
Effects of EPA on PA-induced endothelial cell death. After pretreatment with or without EPA for 2 h, the HUVECs were treated with 0.5 mM PA for 18 h in the presence of 50 μM EPA. Photomicrographs from phase-contrast microscopy (**A**); Cell viability was measured via the MTT assay (**B**); Cell death was determined by LDH release (**C**); Apoptotic cells were evaluated using the terminal deoxynucleotidyl transferase-mediated dUTP nick end-labeling (TUNEL) staining assay (**D**). The data are expressed as the means ± the S.E.s of five independent experiments. ^#^ *p* < 0.05 *vs.* untreated control; * *p* < 0.05 *vs.* PA treatment. Scale bar = 100 µm.

The protective effects of EPA against cell death were first evaluated in HUVECs. As shown in Figure 1A, PA increased the numbers of shrunken cells and the numbers of cells with blebbing membranes, and pretreatment with EPA significantly reduced these phenomena. The MTT assay was used to measure cell viability. The viabilities of HUVECs treated with PA were significantly lower than those of untreated cells. Pretreatment with EPA dose-dependently reduced the PA-induced cell death (Figure 1B). EPA treatment suppressed the PA-induced release of LDH, which is indicative of cell membrane leakage (Figure 1C), in a dose-dependent manner. Additionally, PA-induced cell apoptosis was suppressed by 50 μM EPA pretreatment (Figure 1D).

### 2.2. EPA Inhibited the PA-Induced Intracellular Superoxide Production and ROS Generation

**Figure 2:**
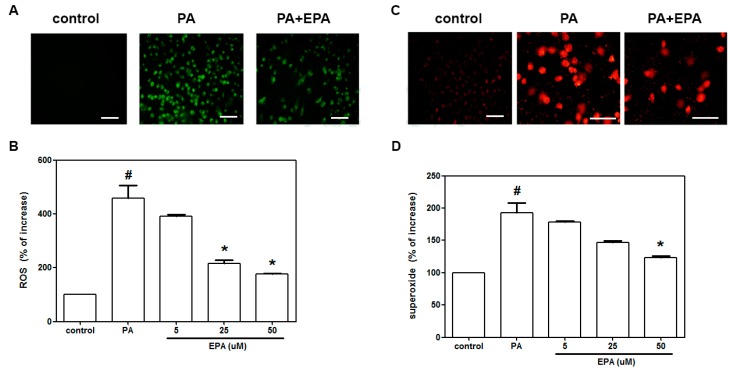
The protective effects of EPA on PA-induced ROS and superoxide generation in HUVECs. After pretreatment for 2 h with the indicated concentrations of EPA (5–50 μM), the HUVECs were incubated with DCF-AM or DHE for 1 h, followed by treatment with PA (0.5 mM) for 2 h in the presence of EPA. Fluorescence images indicating the ROS (**A**) and superoxide levels (**C**) in the controls (**left**) and the cells that were stimulated with PA (**middle**) in the presence of 50 μM EPA (**right**); The fluorescence intensities of the HUVECs were determined using a fluorescence microplate reader. The fluorescence distributions of DCF-AM oxidation (**B**) and DHE (**D**) expressed as percentage increases in intensity. The data are expressed as the means ± the S.E.s of five independent experiments. ^#^ *p* < 0.05 *vs.* untreated control; * *p* < 0.05 *vs.* PA treatment. Scale bar = 100 µm.

To clarify whether the observed anti-apoptotic effect of EPA was associated with a reduction in oxidative stress, we further used DCF-AM staining to measure the generation of ROS. We found that 18 h of exposure to PA produced a five-fold increase in ROS generation. Pretreatment of the HUVECs with EPA (5–50 μM) led to a dose-dependent reduction in ROS generation (Figure 2B). Superoxide radical formation was measured by dihydroethidium (DHE) fluorescence (Figure 2C). A significant increase in constitutive DHE fluorescence was found in the PA-treated cells compared to the control cells (Figure 2D). Treatment with EPA (50 μM) reduced the DHE fluorescence that followed exposure to PA.

### 2.3. EPA Protected against the PA-Induced Apoptotic Response

**Figure 3:**
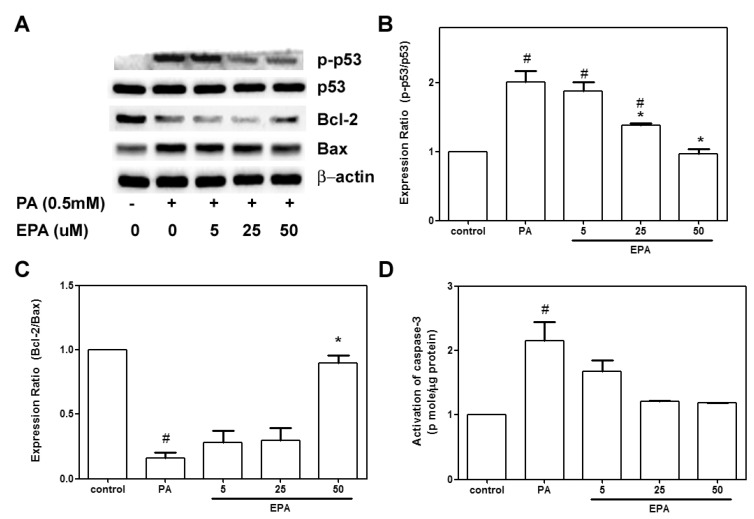
Effects of EPA on the PA-mediated upregulation of apoptosis-related proteins. The HUVECs were pretreated with the indicated concentrations of EPA for 2 h followed by further stimulation with PA (0.5 mM) for another 18 h. The expressions of phosphor-p53, p53, Bcl-2, Bax and β-actin were determined by western blot (**A**); Representative western blots and phosphor-p53/p53 ratio (**B**) and Bcl-2/Bax ratio (**C**) summary data are shown. These results were confirmed by densitometric analyses; Caspase 3 activity was measured with an EnzCaspase-3 assay kit (**D**); The values are presented as the means ± the S.E.s of three separate experiments. ^#^ *p* < 0.05 *vs.* untreated control; * *p* < 0.05 *vs.* PA treatment.

The apoptotic response was further investigated by measuring caspase-3 activity and apoptosis-related proteins with western blot techniques. PA administration caused 2.5- and 2-fold increases in caspase-3 activity and p53 phosphorylation, respectively. Pretreatment with 50 μM EPA led to 100% reductions in caspase-3 activity and phospho-p53 compared to the PA-treated cells. No significant differences were observed in caspase-3 activation or p53 phosphorylation in the HUVECs that were treated with DMSO (negative control) and those that were treated with 50 μM EPA (Figure 3A,C). Additionally, PA significantly decreased Bcl-2 expression and increased Bax expression. The Bcl-2/Bax ratio in the PA-treated group was reduced to 25% of that observed in the control group. Pretreatment with EPA dose-dependently reversed the PA-induced downregulation of Bcl-2 (Figure 3B). 

### 2.4. EPA Suppressed PA-Induced Downregulation of eNOS

**Figure 4:**
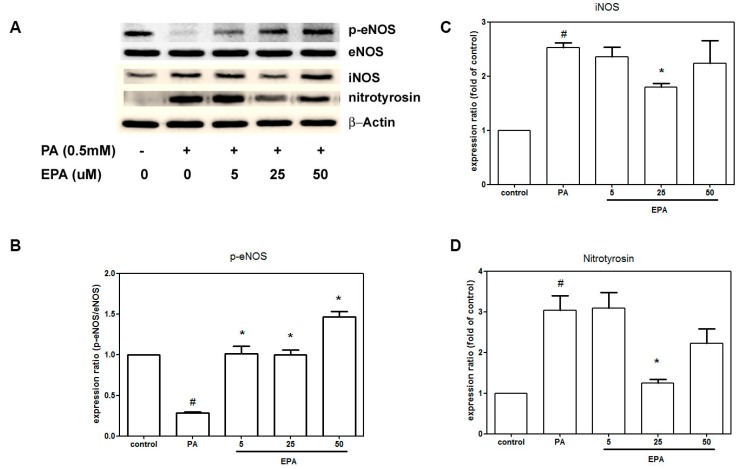
EPA attenuated the PA-induced downregulation of eNOS and upregulations of iNOS and nitrotyrosine. The HUVECs were pretreated for 2 h with the indicated concentrations of EPA followed by stimulation with PA (0.5 mM) for 18 h in the presence of EPA. The levels of the phosphor-eNOS, eNOS, iNOS and nitrotyrosine proteins were determined by western blot (**A**); The quantifications of the related phospho-eNOS (**B**); iNOS (**C**) and nitrotyrosine (**D**) are shown. The values are presented as the means ± the S.E.s of three independent experiments. ^#^ *p* < 0.05 *vs.* untreated control; * *p* < 0.05 *vs.* PA treatment.

A defect in eNOS activation has been proposed to be a major mechanism of endothelial dysfunction. To understand whether NO and NO synthases were involved in the EPA-mediated protection against PA-induced endothelial dysfunction, we explored the effects EPA and PA on the levels of the phospho-eNOS, eNOS, iNOS and nitrotyrosine proteins. Figure 4A shows that exposure to neither PA nor EPA altered the eNOS expression levels. However, PA significantly decreased phospho-eNOS and increased iNOS and nitrotyrosine compared to the negative control. In contrast, pretreatment with EPA increased the expression of phospho-eNOS, which was reduced by PA. Furthermore, the PA-enhanced expressions of iNOS and nitrotyrosine were suppressed by 25 μM EPA.

### 2.5. EPA Attenuated PA-Induced NADPH Oxidase Activation

**Figure 5:**
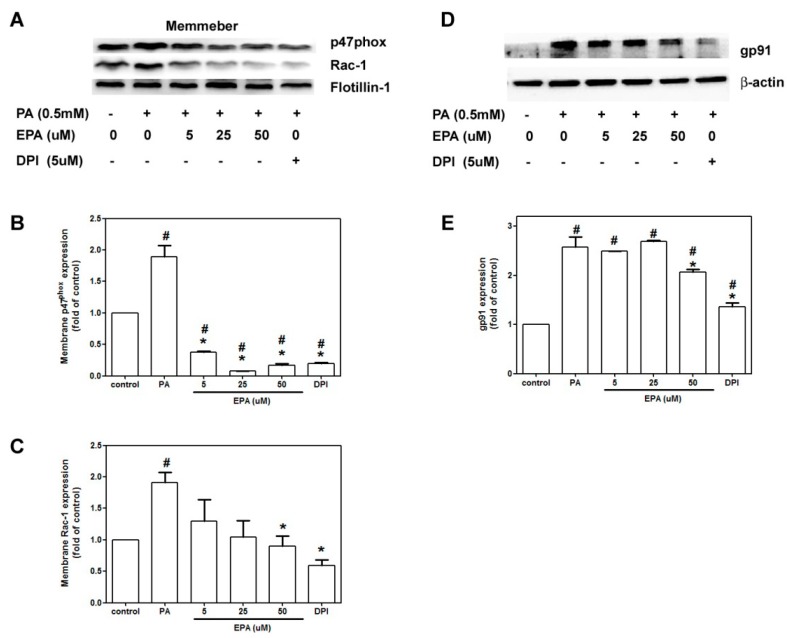
Effects of EPA on PA-induced NADPH oxidase activation. The HUVECs were pretreated with EPA 2 h prior to 18 h of incubation with PA (0.5 mM). The membrane and cytosolic proteins were extracted. Representative immunoblots for p47phox, Rac-1 and Flotillin-1 in the membrane (**A**) and gp91 in the cytosol (**D**) fractions; Densitometric analyses of the expressions of p47phox (**B**); Rac-1 (**C**) and gp91 (**E**) are shown. The values are presented as the means ± the S.E.s of three independent experiments. ^#^ *p* < 0.05 *vs.* untreated control; * *p* < 0.05 *vs.* PA treatment.

A previous study has documented that free fatty acids induce the activation of NADPH oxidase [18]. The cytosolic subunits of NADPH oxidase translocate from the cytosol to the membrane when NADPH oxidase is activated. PA increased gp91phox protein expression 2.5-fold compared to the controls, and EPA treatment prevented this effect (Figure 5D). The PA treatments stimulated the translocation of Rac-1 from the cytosol to the membrane, and EPA reduced this effect (Figure 5C). Surprisingly, PA significantly increased the translocation p47phox, and EPA prevented this translocation to the extent that translocated p47phox levels were lower than those in the controls (Figure 5B). 

### 2.6. EPA Protected against PA-Induced Downregulation of eNOS and AKT via AMPK

**Figure 6:**
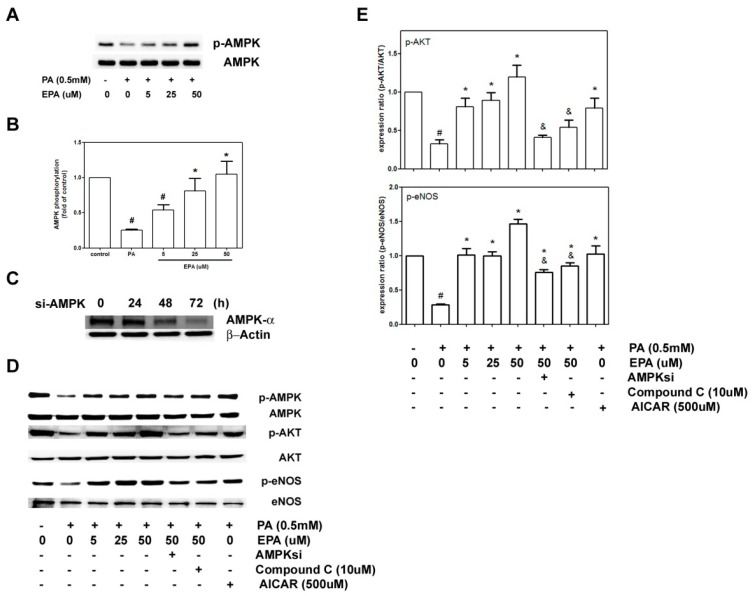
Effects of EPA on PA-induced AMPK activation. (**A**) The HUVECs were pretreated with EPA for 2 h and subsequently treated with 0.5 mM PA for 18 h. Phospho-AMPK and AMPK expression were subjected to western blot analyses; (**B**) The quantifications of the related phospho-AMPK and total AMPK are shown; (**C**) The cells were transiently transfected with AMPKα1 siRNA for 24, 48 or 72 h. The expression levels of phospho-AMPK and total AMPK in the transfected HUVECs were determined by western blot analyses; (**D**) The HUVECs were transfected with AMPKα1 siRNA for 72 h or treated with the AMPK inhibitor compound C or the activator AICAR 1 h prior to incubation for an additional 18 h with PA (0.5 mM). Phospho-eNOS, eNOS, phosphor-AKT and AKT levels were determined by immunoblotting; (**E**) The quantifications of the related phospho-eNOS, eNOS, phosphor-AKT and AKT are shown. The values are presented as the means ± the S.E.s of three independent experiments. ^#^ *p* <0.05 *vs.* untreated control; * *p* < 0.05 *vs.* PA treatment; and ^&^ *p* < 0.05 *vs.* PA + 50 μM EPA.

To clarify whether AMPK played roles in the observed protective effects of EPA on eNOS, which was downregulated by PA, we analyzed the activity of AMPK in the HUVECs that were treated with 0.5 mM PA for 18 h. As shown in Figure 6A,B, the phosphorylation of AMPK was reduced by 70% in the cells that were exposed to PA; in contrast, pretreatment of the HUVECs with EPA (5–50 μM) significantly potentiated the activity of AMPK in a dose-dependent manner. 

We hypothesize that EPA protected against PA-induced endothelial dysfunction via the AMPK pathway. Thus, we further investigated the role of AMPK in the downregulation of AKT and eNOS with AMPK siRNA, the inhibitor compound C and the activator AICAR. After transfection with AMPK siRNA for 72 h, we observed the maximum reduction in AMPK-α. As shown in Figure 6C,D, both the AMPK inhibitor compound C and AMPK siRNA significantly inhibited the protective effects of 50 μM EPA against PA-induced eNOS and AKT downregulation. Similar to the protective effects of EPA, the administration of the AMPK activator AICAR blocked the reduction in p-AKT and p-eNOS induced by PA stimulation. 

### 2.7. EPA Suppressed PA-Induced Activation of p38 MAPK and NF-κB

**Figure 7:**
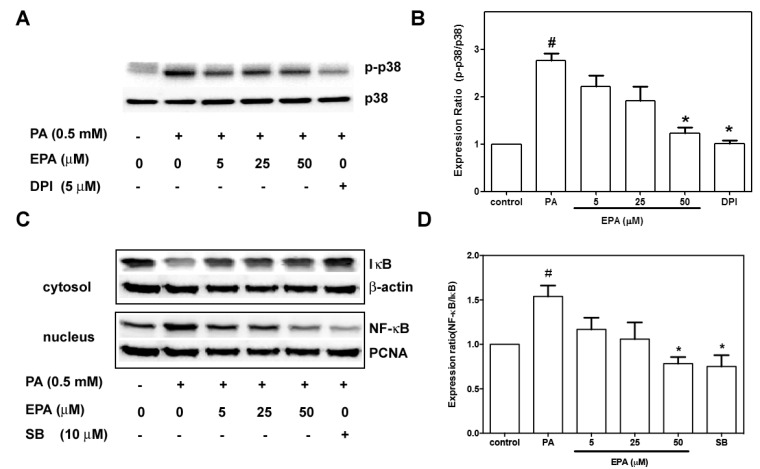
Effects of EPA on PA-induced p38 MAPK and NF-ĸB activation. (**A**) The HUVECs were pre-treated with EPA for 2 h or with the NADPH oxidase inhibitor DPI for 30 m and subsequently treated with 0.5 mM PA for 18 h. Phospho-p38 MAPK and p38 MAPK expression were subjected to western blot analyses; (**B**) The quantifications of the related phospho-p38 MAPK and total p38 MAPK are shown; (**C**) The HUVECs were pretreated with EPA for 2 h or with the p38 MAPK inhibitor SB 203580 for 30 m and subsequently treated with 0.5 mM PA for 18 h. Cytosolic IκB and nuclear NF-κB were determined by immunoblotting; (**D**) The quantifications of the relevant cytosolic IκB and nuclear NF-κB are shown. The values are presented as the means ± the S.E.s of three independent experiments. ^#^ *p* < 0.05 *vs.* untreated control; * *p* < 0.05 *vs.* PA treatment.

Activation of p38 MAPK is involved in endothelial apoptosis [19], and p38 MAPK activation might be mediated through the NADPH oxidase pathway [20]. To understand the effects of EPA on p38 MAPK signals, the phosphorylation of p38 MAPK was determined by immunoblotting. Figure 7A,B shows that exposure to PA or EPA did not change the expression levels of p38 MAPK. Compared to the controls, PA significantly increased phospho-p38 MAPK, and this effect was significantly attenuated by pretreatment with EPA. In contrast, pretreatment with DPI or NADPH oxidase inhibitor reduced the PA-induced phosphorylation of p38 MAPK. 

Previous studies have shown that free fatty acids induce inflammation in the endothelium via the NF-κB pathway [21]. NF-κB proteins are activated by release from a complex they form with IκB in the cytoplasm and subsequent translocation to the nucleus. PA enhanced IκB degradation in the cytoplasm and increased NF-κB translocation to the nucleus (Figure 7C,D). Pre-exposure to EPA or SB203580, a p38 MAPK inhibitor, resulted in significant reductions in PA-stimulated IκB degradation and NF-κB translocation. 

## 3. Discussion

**Figure 8:**
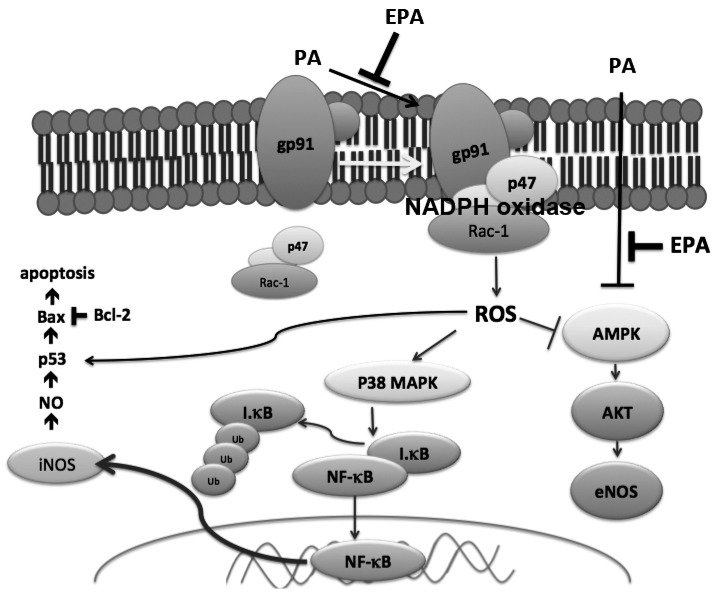
Proposed model of the protective effects of EPA against the PA-induced signaling pathway activation that leads to endothelial dysfunction.

In the present study, we demonstrated that EPA protected against PA-induced endothelial dysfunction by activating AMPK. Our results show that PA suppressed the phosphorylation of AMPK and increased assembly of NADPH oxidase, followed by superoxide generation and downregulation of Akt/eNOS signaling. In addition, PA induced p38 MAPK-mediated NF-κB activation, which is upstream of iNOS. EPA ameliorated these harmful effects through modulating oxidative stress and the AMPK-mediated signaling pathway (Figure 8).

The generation of ROS and superoxide is the key mediator of damage to endothelial cells in atherosclerosis. NADPH oxidase-derived superoxide is the major source of ROS in the endothelium [22]. In hyperglycemia, AMPK reduces NADPH oxidase activation and ROS generation [23], and activation of AMPK can increase the antioxidant MnSOD, which inhibits high glucose-induced intracellular and mitochondrial ROS production [24]. Additionally, AMPK suppresses the resistin-induced membrane assembly of NADPH oxidase and increases eNOS activity in endothelial cells [25]. We found that PA-induced the translocations of p47 phox and Rac-1, and AMPK suppression was abolished in the cells that were pretreated with EPA, which indicates that EPA attenuated oxidative stress and downregulation of AMPK.

Endothelial apoptosis plays an important role in the development of atherosclerosis, and palmitate induces apoptosis in mouse aortic endothelial cells [26]. Chai and Liu [27] observed PA-induced apoptosis that was mediated by p38 MAPK in endothelial cells. PA stimulates the generation of ROS in L6 skeletal muscle cells and leads to activation of caspase-3, which subsequently induces DNA damage and apoptosis [28]. Our results showed that EPA significantly reduced cell apoptosis, the activation of caspase-3 and the expression of Bax. In contrast, EPA also increased the expression of the anti-apoptotic protein Bcl-2 (Figure 3). Combined with its inhibitory effects on ROS generation, we speculate that EPA protected the HUVECs against PA-induced apoptotic responses via anti-oxidation. 

The ability to generate NO has served as a marker for healthy endothelia. Endothelial-derived NO is produced by eNOS and regulates vascular tone. Studies have shown that nanomolar concentrations of NO have anti-inflammatory and protective effects that are mediated by the inhibition of the activation of NF-κB [29]. In contrast, iNOS led to inflammatory responses via NF-κB, which is the key transcription factor for major proinflammatory cytokines and adhesion molecules [30]. Our results show that PA inhibited AKT and eNOS phosphorylation and increased iNOS expression and that all of these effects were ameliorated by pretreatment with EPA. Additionally, pretreatment with compound C, a selective AMPK inhibitor, or the introduction of AMPK siRNA antagonized the effects of EPA on the activation of eNOS and AKT. In contrast, pretreatment with the AMPK activator AICAR significantly restored AKT and eNOS levels, and these effects were inhibited by PA. These indicated that EPA increased eNOS phosphorylation through AMPK/AKT signaling pathway. Furthermore, this upregulation of eNOS by AMPK is consistent with the finding that EPA can increase eNOS activity via AMPK *in vivo*. Furthermore, both AMPK and eNOS are required for the EPA-mediated endothelial protection against EPA in a mouse model of atherosclerosis [31]. These results suggest that the AMPK/AKT/eNOS/NO signaling pathway plays a role in the protective effects of EPA. 

Local inflammation contributes to the progression of atherosclerosis [32], and EPA can reduce free fatty acid-induced inflammation [33]. There is evidence that activation of the p38 MAPK/NF-κB pathway occurs with increases in oxidative stress, which indicates that the p38 MAPK/NF-κB pathway might be downstream of NADPH oxidase [34]. Our results showed that EPA significantly reduced the activation of p38 MAPK and suppressed the degradation of IκB and the translocation of NF-κB, which plays a critical role in the expression of pro-inflammatory cytokines. Our findings that the NADPH oxidase inhibitor reduced PA-induced activation of p38 MAPK and that inhibitors of p38 MAPK blocked PA-induced p38 MAPK phosphorylation clearly indicate that the protective effects of EPA were due to the modulation of the NADPH oxidase/p38MAPK/NF-κB pathway. 

In summary, we have demonstrated that EPA protects against PA-induced oxidative stress and apoptosis by activating AMPK signaling. Our results provide insight into some of the mechanisms by which EPA protects against endothelial damage.

## 4. Experimental Section

### 4.1. Materials

Anti-flotillin-1, anti-phospho-AMPK-α, anti-AMPK-α, anti-IκB, anti-NF-κBα, anti-p27, anti-AKT, anti-Bax, anti-Bcl, and anti-p38MAPK antibodies were obtained from Cell Signaling Technology (Danvers, MA, USA). Anti-p22 antibody was obtained from Abcam Inc. The TUNEL staining kit was purchased from Boehringer Mannheim (Mannheim, Germany). The EnzChek caspase-3 assay kit was obtained from Molecular Probes (Eugene, OR, USA). Anti-eNOS, anti-phospho-eNOS, anti-iNOS, anti-nitrotyrosine, anti-P53, anti-phospho-P53, anti-Bcl2, anti-gp91, anti-p22phox, and anti-Bax were obtained from Transduction Laboratories (San Diego, CA, USA). AMPK-α1-specific small interfering RNA (siRNA) (sc-29673) was purchased from Santa Cruz Biotechnology (Santa Cruz, CA, USA). EPA, SB203580, and diphenylene iodonium (DPI) were obtained from Sigma-Aldrich (St. Louis, MO, USA). 

Palmitic acid was obtained from Alltech Associates Inc. and dissolved in 0.1 mol/L NaOH at 70 °C and then complexed with 10% BSA at 55 °C for 10 min to achieve the final palmitate concentration. EPA (>99% purity, Sigma–Aldrich, St. Louis, MO, USA, cat # E2011) was dissolved in ethanol at the designated concentrations. Equivalent amounts of solvent were added to the control cells. 

### 4.2. Cell Cultures

This study was approved by the Research Ethics Committee of the China Medical University Hospital. 

Human umbilical vein endothelial cells (HUVECs) were isolated from human umbilical cords with collagenase and were used at passage 2 to 3 as described previously [35]. Briefly, after dissociation, the cells were collected and cultured on gelatin-coated culture dishes in medium 199 with low serum growth supplement, penicillin (100 IU/mL), and streptomycin (0.1 mg/mL). Subcultures were performed with trypsin-ethylenediaminetetraacetic acid (EDTA). The media were refreshed every 2 days.

### 4.3. Determinations of Cell Viability, Cytotoxicity and Apoptosis

To determine the effects of EPA on PA-induced cytotoxicity, HUVECs were incubated with the indicated concentrations of EPA for 2 h and then exposed to 0.5 mM PA for an additional 18 h. Cell viability was assessed with the MTT assay [36], and cell death was determined with the LDH release assay [37]. Apoptotic cells were identified by TUNEL assay and visualized under a fluorescence microscope [38]. 

### 4.4. Measurement of Superoxide and ROS Production

The effects of EPA and PA on intracellular ROS in the HUVECs were determined by fluorometric assays that used dihydroethidium (DHE; Sigma, St. Louis, MO, USA) and DCF-AM (Invitrogen, Carlsbad, CA, USA). DCF-AM was used as a general probe for ROS; DHE was used as a specific probe for superoxide [39]. HUVECs (10^4^ cells/well) were seeded in 24-well plates, pre-incubated with various concentrations of EPA for 2 h, and then exposed to 0.5 mM PA for another 18 h. The cells were then exposed to 10 μM DHE or DCF-AM for 1 h. Fluorescence intensity was measured with a fluorescence microplate reader (Labsystems, Mountain View, CA, USA) that was calibrated for excitation and emission wavelengths. For DHE, excitation and emission wavelengths are 530 and 590 nm. For DCF-AM, excitation and emission wavelengths are 485 and 538 nm.

### 4.5. Western Blot Analyses

HUVECs were grown to confluence, pretreated with various concentrations of EPA for 2 h, and then stimulated with PA (0.5 mM) for 18 h. After treatment, membrane and cytosolic extracts were prepared using MEM-PER nuclear and cytoplasmic extraction reagents (Pierce, Rockford, IL, USA) according to the manufacturer’s recommendations. The expression levels of p-AMPKα, AMPKα, p-eNOS, eNOS, p-p53, p53, Bcl-2, Bax, iNOS, and nitrotyrosine in the cytosolic fractions and those of p47phox, Rac-1, and gp91phox in the membrane fractions were determined by SDS-PAGE and immunoblot assays. The signals were quantified with ImageJ software (National Institutes of Health, Bethesda, MD, USA) as described previously [40].

### 4.6. Transfection with Small Interfering RNAs (siRNAs)

AMPKα1 siRNAs were purchased from Santa Cruz Biotechnology (Santa Cruz, CA, USA). Transient transfection was carried out using INTERFERin siRNA transfection reagent (Polyplus Transfection, New York, NY, USA) according to the manufacturer’s instructions. Three days after transfection, the cells were treated with reagent as indicated for further experiments.

### 4.7. Measurement of Active Caspase-3

To explore the effects of EPA on PA-induced caspase-3 activation, HUVECs were pretreated with EPA for 2 h and then stimulated with PA (0.5 mM) for 18 h. The levels of active caspase-3 were detected with an EnzChek caspase-3 assay kit according to the manufacturer’s instructions (Invitrogen Life Technologies; Carlsbad, CA, USA). Fifty micrograms of protein was added to a reaction buffer containing 5 mM of the caspase-3 substrate Z-DEVD-R110, and the mixture was incubated at room temperature for 30 min. The fluorescence generated from the cleavage of the substrate by caspase-3 was monitored with a fluorescence microplate reader (Labsystems, Mountain View, CA, USA) calibrated for excitation and emission wavelengths of 496 and 520 nm, respectively.

### 4.8. Statistical Analyses

The results are expressed as the mean ± the SEMs. The differences between groups were analyzed with one-way analyses of variance followed by Bonferroni’s *post hoc* tests. A *p*-value < 0.05 was considered to represent statistical significance.
